# Bioactivity of indigenous medicinal plants against the cotton whitefly, *Bemisia tabaci*

**DOI:** 10.1093/jis/14.1.105

**Published:** 2014-08-01

**Authors:** E. Abou-Fakhr Hammad, A. Zeaiter, N. Saliba, S. Talhouk

**Affiliations:** 1 Department of Agricultural Sciences, Faculty of Agricultural & Food Sciences, American University of Beirut, Beirut, Lebanon; 2 Al Ryum Contracting Company, Doha, Qatar; 3 Department of Chemistry, Faculty of Arts & Sciences, American University of Beirut, Beirut, Lebanon; 4 Department of Landscape Design & Ecosystem Management, Faculty of Agricultural & Food Sciences, American University of Beirut, Beirut, Lebanon

**Keywords:** plant extract, medicinal, endemic

## Abstract

Forty-one methanol extracts of 28 indigenous medicinal plant species were tested for their insecticidal bioactivity against cotton whitefly, *Bemisia tabaci* (Gennadius) (Hemiptera: Aleyrodidae), adults and second nymphal instars under controlled conditions. This study is within a bioprospection context, in the form of utilizing local plant species as an alternative in sustainable agriculture development. Eighteen and nine plant extracts caused a significant decrease in number of live adult and nymphal whiteflies, respectively, compared to the control. This is the first report for the potential effect on survival of insects for 22 out of 28 tested medicinal plant species. Whole plant extracts of *Ranunculus myosuroudes* Boiss. and Kotschy (Ranunculaceae), *Achillea damascena* L. (Asteraceae), and *Anthemis hebronica* Boiss. and Kotschy (Asteraceae) and leaf extracts of *Verbascum leptostychum* DC. (Scrophulariaceae) and *Heliotropium rotundifolium* Boiss. (Borangiaceae) caused both repellent and toxic effects against the adult and second nymphal instars, respectively. Extracts of leaves and stems of *Anthemis scariosa* Boiss. (Asteraceae) and *Calendula palestina* Pers. (Asteraceae) were found to be more bioactive against the adult and nymphal instars, respectively, than extracts of other plant parts, such as flowers. Thus, the bioactive extracts of these medicinal plants have the potential to lower whitefly populations in a comprehensive pest management program in local communities, pending cultivation of these medicinal plant species.

## Introduction

The cotton whitefly, *Bemisia tabaci* (Gennadius) (Hemiptera: Aleyrodidae), has been recorded from more than 600 different plant species, and its polyphagous nature has been documented worldwide ( Cock 1986 ; Greathead 1986 ). Common insecticidal control of *B. tabaci* on crops consists predominantly of foliar-applied sprays of active ingredients that are dependent on spray coverage and deposition ( Palumbo et al. 2001 ). In many cropping systems, repeated spray applications have been necessary and often result in overuse of these chemicals. Consequently, *B. tabaci* has developed resistance to numerous conventional insecticides throughout the world ( Dittrich and Ernst 1990 ; Gerling and Kravchenko 1995 ; Palumbo et al. 2001 ). These problems have increased the need for effective, biodegradable pesticides with greater selectivity and alternative strategies that include the search for new types of insecticides and the reevaluation and use of traditional botanical pest control agents (Anon. 2003).

Many plants have developed chemical defenses to deter herbivores that eat them. These plants may be cultivated to provide sources of biodegradable pesticides ( Farombi 2003 ). The world market for insecticides is large, and consumer preferences for natural over synthetic pesticides are growing. Many indigenous plants are used locally for herbal and medicinal purposes. Indigenous medicinal plants are relevant in both developing and developed nations of the world as sources of drugs or herbal extracts for various chemotherapeutic purposes ( Purbrick 1998 ; Farombi 2004 ).

A few studies have dealt with the use of medicinal plants or their components as potential pesticides against whiteflies. Hilje at al. (2003) tested 70 plant extracts in Costa Rica for repellency or deterrence against *B. tabaci* adults. Extracts from 10 medicinal plant species showed the ability to deter or repel adult whiteflies. Their effect has been detected under greenhouse experimental conditions at doses as low as 10 mL/L water (1% v/v) ( Hilje et al. 2003 ). Ateyyat et al. (2009) also tested for the toxicity of aqueous extracts of nine plants known to have medicinal activity against the sweet potato whitefly and compared them to the toxicity of the insecticide Imidacloprid. Extracts of *Lepidium sativum* L. (Brassicales: Brassicaceae) killed 71% of early-stage nymphs, which was not significantly different from mortality caused by Imidacloprid. Treatment of pupae with the three plant extracts *L. sativum, Achillea biebersteinii* L. (Asterales: Asteraceae), and *Retama raetam* (Forssk.) Webb and Berthel (Fabales: Fabaceae) prevented adult development. Treatment with *R. raetam* extract killed adults at levels that were not significantly different from Imidacloprid. However, extracts of four plants, *Pimpinella anisum* L. (Apiales: Apiaceae) *, Galium longifolium* (Sibth. and SM.) (Gentia-nales: Rubiaceae), *R raetam,* and *Ballota undulata* Bentham (Lamiales: Lamiaceae) had a repellent effect.

Fenigstein et al. (2001) found that deterrence of five economically important vegetable seed oils (peanut, cottonseed, castor, soybean, and sunflower) was strong enough to cause adult death of *B. tabaci* due to starvation or dehydration under no-choice conditions in a laboratory study. Choi et al. (2003) studied 53 plant essential oils for their insecticidal activity against the greenhouse whitefly, *Trialeurodes vaporariorum* Westwood. Oils from 9 out of 53 plant species were highly effective as a result of action in the vapor phase. Responses varied according to oil type and dose and developmental stage of the insect.

Preserving indigenous plants can be a sustainable means for livelihood, especially if they are incorporated into economical and feasible agricultural practices such as pest management with extracts of local plant species. The structurally diverse natural compounds of these extracts might act as deterrents, antifeedants, growth inhibitors, and toxoids. The main objective of this study is to screen 28 medicinal plant species native to Lebanon for their activity against the adult and nymphal instars of the whitefly, *B. tabaci* , which has developed high resistance to conventional chemical control methods.

## Materials and Methods

### Plant extract preparation

Plant selection, collection, and extraction were performed according to a procedure set for testing different bioactivities of plant extracts at the Center of Initiative for Biodiversity Studies in Arid Regions (IBSAR), located on the American University of Beirut premises.

### Plant material.

A list of 318 plant genera native to Lebanon with potential medicinal and/or agricultural uses was generated at IBSAR center. The initial holistic plant list of 399 species was based on five prior bioactivity categories: antitumor, anti-inflammatory, analgesic, antimicrobial, and insecticidal. Out of the holistic list, 109 plants with insecticidal usages have been detected; this indicates that 27.3% of total plants revised for all above categories have insecticidal bioactivity. The selected tested plant species were all indigenous to Lebanon; the plant list included 28 plant species ( Table 1 ).

**Table 1.**
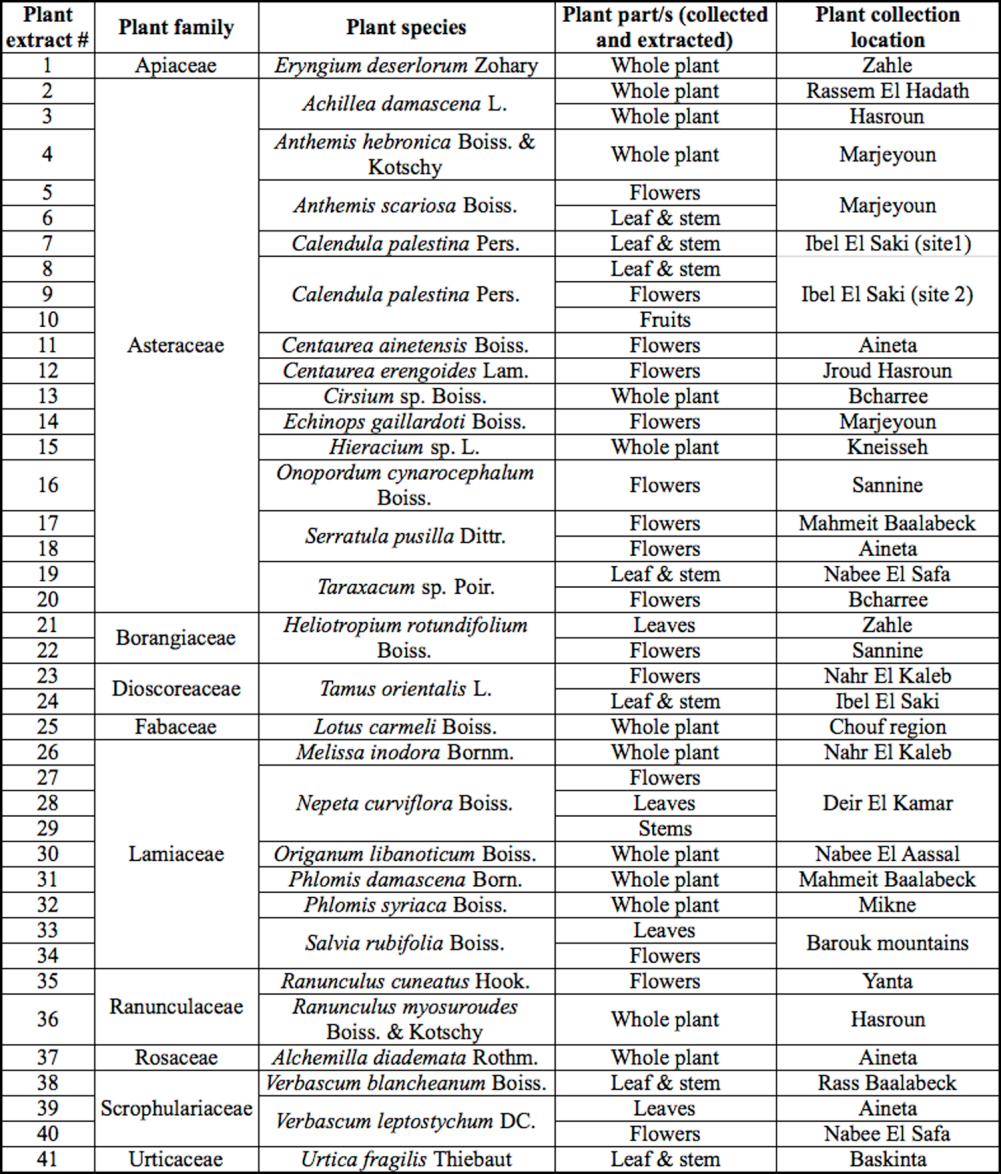
List of extracts of indigenous medicinal plant species tested for their insecticidal bioactivity against adults and 2 ^nd^ instar nymphs of *Bemisia tabaci.*

Plants belonging to 10 botanical families were collected between March and August 2002, during which plant species were harvested for their leaves, flowers, fruits, or stems as chemical composition of plant extracts varies with the part of the sample studied ( Banthrope and Charlwood 1980 ). Forty-one extracts of 28 plant species were tested for their insecticidal bioactivity in this study ( Table 1 ).

### Extraction method of plant material.

Extraction of plant parts was performed at the Department of Chemistry in the Faculty of Arts & Sciences, American University of Beirut, at room temperature.

Harvested plants were washed with distilled water to remove any contaminants on the surface of plant parts and were dried in the shade at a temperature of 25-32ºC with adequate ventilation for two weeks. They were broken down physically into flowers, leaves, and stems, or kept as a whole plant, including leaves, flowers, and stems (in cases of low availability of the plant species). Plant parts were ground into very small particles (0.3 mm in diam.) in a mill (SM 100 Cutting Mill, Brinkmann, Germany) at a speed of 1,600 rpm at 60 HZ.

Powdered ground material of each plant species was soaked in methanol for 16 hr and placed in a shaker-incubator for the first 2 hr at room temperature. Methanol was used to extract most of the semi-polar and polar constituents. This pure MeOH extraction was performed using the standard plant material/solvent concentration of 1:10 (w:v); the extract was filtered by vacuum pressure through several layers of sterile cheesecloth. Filtrate of each raw extract was stored in 10 mL or 5 mL glass vials wrapped with aluminum foil at -20ºC for use in the bioassays.

### Screening bioassays with plant extracts against *B. tabaci.*

All experiments were performed under controlled conditions in a glasshouse at the Faculty of Agricultural & Food Sciences, American University of Beirut, at 25 ± 2°C, 80 ± 10% RH, and 16L:8D photoperiod. Treatments included 41 extracts of 28 plant species with two controls: methanol (10%) and distilled water. Each treatment was replicated three times on one date of the experiment and nine times within different batches during the study. Due to the availability of consistent controlled conditions during the experiments with whitefly adults and nymphs, 14-15 treatments were performed simultaneously on different days of the experiment, allowing 42-45 cages to be occupied with treated seedlings on each date, with one seedling per cage.

### Whitefly colony.

A *B. tabaci* colony was raised in a glasshouse compartment under controlled conditions as mentioned above. The colony, originally from a field population, was reared on cucumber plants, *Cucumis sa-tivus* L. (Cucurbitales: Cucurbitaceae) of the variety Beit Alpha (F1 parthenocarpic, dust free and thiram treated seeds; Edena Seeds, USA) in a whitefly-proof cage (140 x 85 x 130 cm) covered completely with a fine mesh (270 x 770 um) *.* Two true leaf seedlings were grown in 12 cm plastic pots to provide a continuous supply of healthy young plants to the whitefly colony and bioassays. Fertilization with Floral® (20-20-20+ microelements; Cifo S.p.A., Bologna, Italy) was applied at a rate of 5g per 10 L through irrigation about two times per week.

### Experimental setup with whitefly adults.

Cucumber seedlings, having two true leaves with detached cotyledons, were used in the bioassays. Using a rotary evaporator, 10 mL of each methanol extract was reduced to 1 mL, after which 9 mL of distilled water was added to homogenize the solution before application to the plant. Each plant received an average of 9 mL of the extract or the control (distilled water or 10% methanol), sprayed on the upper and lower sides of the leaves with 10-mL glass-bottle sprayers. Each treated seedling was allowed to air-dry and then placed in a plastic cage (28 cm high x 21 cm diam., manufactured locally) with a 15 cm aeration opening at the top and two 5 cm circular openings in the body of the cage covered with 270 x 770 μm mesh.

Ten adult whiteflies (about three days old) were collected by hand aspirator (Hausherr’s machine, N.J., USA) and introduced into each plastic cage after treatment. Numbers of adult whiteflies (dead or alive) in each cage were recorded at 72 hr after treatment, noting the location of the insect in the cage: on the plant, walls, or top cover of the cage, or on the soil surface in the pot. Selection of repellency assessment at 72 hr after treatment was based on the following observations related to the extracts. A few hours after treatment, adult whiteflies were found landing at the top of the cage; this could be attributed to the fact that the bioactive extracts contained some volatile compounds that repelled the insects from approaching the plant. However, observations at 24 hr detected the presence of live whitefly adults on the treated plant, which indicated no direct toxicity effect for some extracts on *Bemisia* adults. Thus, observations at 72 hr confirmed the repellent efficacy of some extracts on whiteflies, knowing that botanicals are in general characterized by reduced stability, which indicates that the presence of a pest on treated leaves was more obvious with time ( Sundaram 1996 ), especially with nonrepellent extracts.

### Experimental setup with whitefly nymphs.

Cucumber seedlings, having two true leaves with detached cotyledons, were placed in plastic cages as described above. Ten adult whiteflies (about three days old) collected from the rearing colony were introduced into each cage in a vial and were allowed to oviposit for two days to limit the number of eggs laid. After 8–10 days for the introduction of adult whiteflies, 40 second-instar nymphs were counted using a stereomicroscope and were circled on the lower leaf surface. Nymphs other than second instars were removed using a fine brush. Plant extracts to be applied were prepared in a manner similar to the previous procedure with the adult whiteflies. Each plant was sprayed on both sides of nymph-infested leaves, kept to dry, and then transferred to a cage. After 10 days of treatment, total number of nymphs was recorded according to each developmental stage (either second or third instar) by cutting the leaf from the plant and observing the status of nymphs under the stereomicroscope: healthy, dry, or oozing.

### Statistical analysis.

Trials were laid out in a completely randomized block design with two factors: treatment and date of batch. Each treatment was repeated on three different dates, with each treatment replicated three times on each date. As there was no significant interaction among the two factors, the data were pooled and analyzed as a one-way ANOVA, with the treatment as the main factor. Consequently, there were 43 treatments (including two controls) with nine replicates per treatment. The average numbers of live whitefly adults or nymphs per plant were used in the data analysis, after ensuring their normal distribution by transforming the data using sqrt ( *x* + 1), with *x* being the number of live whitefly adults or nymphs per plant. The analysis of data was performed using the general linear model procedure, and means were separated by Student-Newman-Keuls test whenever *P* <.05 was detected ( SAS 1992 ); this test provides different critical difference values for particular comparisons of means depending on how adjacent the means are ( Stevens 1999 ).

The treatments were divided into seven groups for simplification of the data analysis; data for each treatment group that included plant extracts and the two controls were statistically analyzed separately. The four major treatment groups included 15 flower extracts, 13 whole plant extracts, seven leaf and stem extracts, and four leaf extracts. Data for the two remaining extracts, one fruit extract and one stem extract, were studied in a comparative analysis for the data pertaining to different plant parts of the same plant species collected from one location; this treatment group included 10 extracts. Another treatment group dealt with effects of plant source (collection location) on bioactivity of the extracts; this treatment group included six extracts. A further treatment group dealt with effects of similar plant parts of different species within one plant genus on bioactivity of the extract; this treatment group included four extracts.

## Results

Results of our bioassays show efficacy in decreasing the encountered number of live adult and nymphal whiteflies in some treatments with medicinal plant extracts under glasshouse conditions, according to data analysis of the seven treatment groups mentioned above.

In the first treatment group, there was significant difference in the number of live whitefly adults (F = 4.30; df = 16, 152; *P* <.0001) and nymphs (F = 2.68; df = 16, 152; *P* <.0011) among treatments with 15 flower extracts of different plant species (including only one plant species, *Serratula pusilla* Dittr., collected from two locations, Table 1 ) and the two controls ( Table 2 ). Flower extracts of three plant species, *Tamus orientalis* L. *, S. pusilla* (collected from labeled location 1, Table 1 ), and *Onopordum cynarocephalum* Boiss., were significantly different in the number of live adult whiteflies after treatment compared to the two controls. The former extract seemed to have the highest repellent effect compared to all other treatments, with the lowest number of whiteflies (1.00 adult whitefly/plant). For nymphs, flower extract of *Echinops gaillardoti* Boiss. was significantly different in the number of live nymphs encountered after treatment compared to the two controls. This extract had the lowest count of 18.22 nymphs/plant, and seemed to have the highest toxic effect to the whitefly nymphs compared to all other treatments in this group. All other extracts were not significantly different in number of live adults or nymphs after treatment compared to the MeOH(10%) and/or water controls.

**Table 2.**
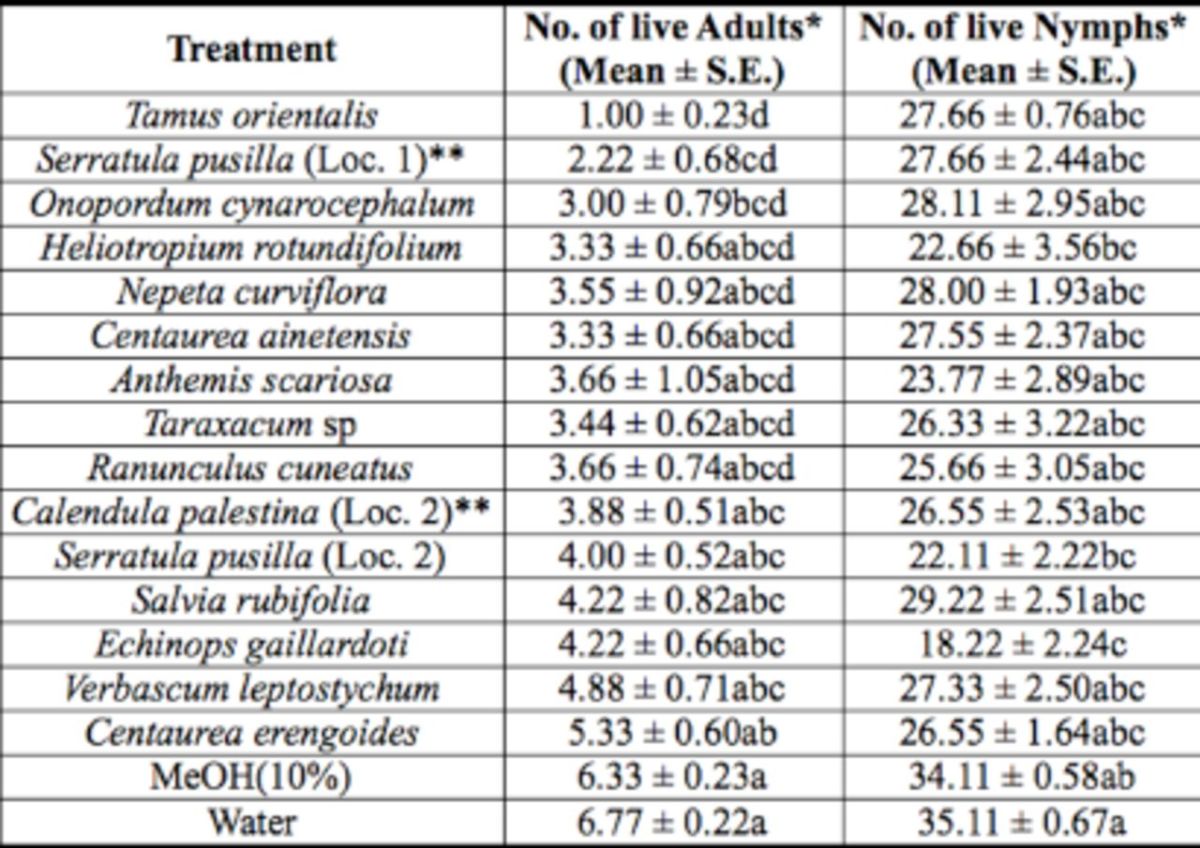
Number of *B. tabaci* adults and nymphs encountered live on treated cucumber plants with methanol flower extracts of different plant species under controlled glasshouse conditions.

Measurement values presented are mean ± SEM.

*Means in a column followed by different letters (a/b/c/d) are significantly different (Student- Newman-Keuls test, *P* <.05).

**Loc. 1 or 2 refers to location number from where plant species was collected.

In the second treatment group, there was a significant difference in the number of live whitefly adults (F = 6.19; df = 14, 134; *P* <.0001) and nymphs (F = 4.09; df = 14, 134; *P* <.0001) among treatments with 13 whole plant extracts of different plant species and the two controls ( Table 3 ). Eight out of 13 whole plant extracts were significantly different in the number of live adults encountered after treatment compared to the two controls. For nymphs, four out of 13 whole plant extracts were significantly different in the number of live nymphs encountered after treatment compared to the two controls. However, these extracts were not significantly different from each other in the number of live adults/nymphs present. The whole plant extract of *Ranunculus myosuroudes* Boiss. and Kotschy had the lowest count (1.22 adult whiteflies/plant and 16.66 nymphs/plant), and seemed to have the highest repellent and toxic effects to the whitefly adults and nymphs, respectively, compared to all other treatments in this group.

**Table 3.**
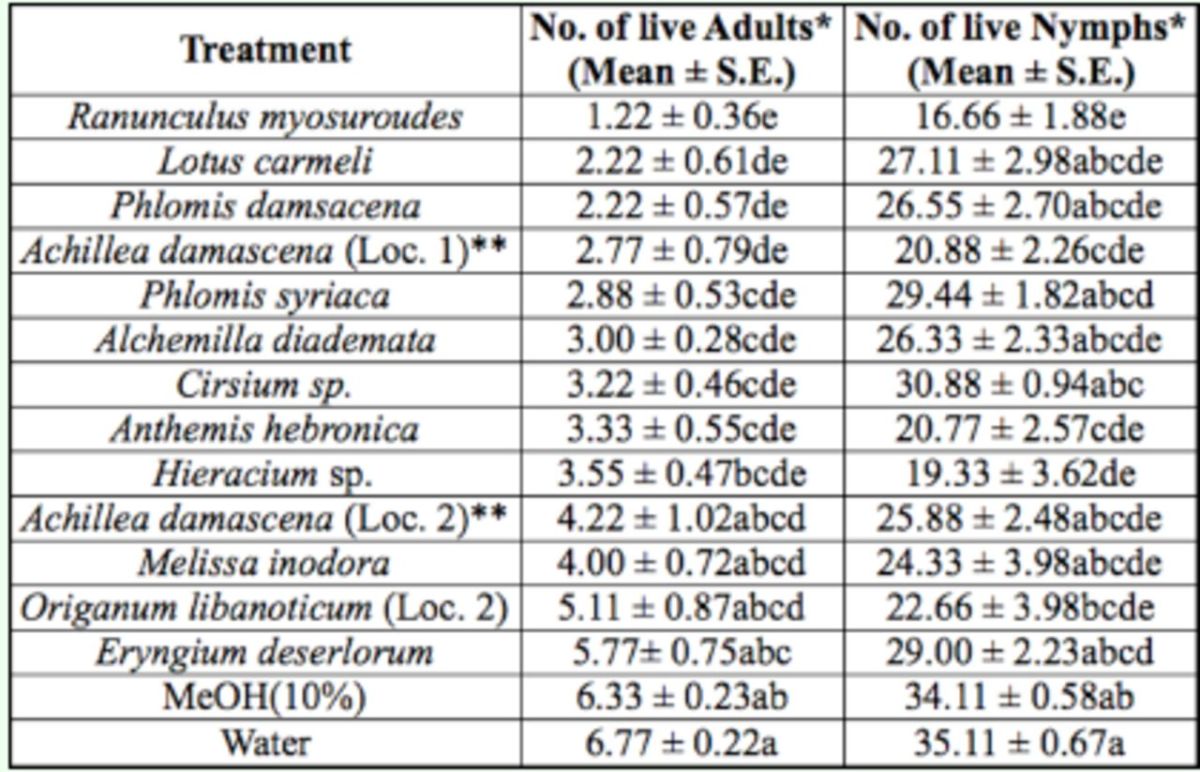
Number of *B. tabaci* adults and nymphs encountered live on treated cucumber plants with methanol whole plant extracts of different plant species under controlled glasshouse conditions.

Measurement values presented are mean ± SEM.

*Means in a column followed by different letters (a/b/c/d/e) are significantly different (Student- Newman-Keuls test, *P* <.05).

**Loc. 1 or 2 refers to location number from where plant species was collected.

In the third treatment group, there was a significant difference in number of live whitefly adults (F = 4.40; df = 8, 80; *P* = 0.0002) and nymphs (F = 5.15; df = 8, 80; *P* <.0001) among treatments with seven leaf and stem extracts of different plant species, including one plant species, *Calendula palestina* Pers., collected from two locations ( Table 1 ), and the two controls ( Table 4 ). Leaf and stem extracts of three plant species, *Anthemis scariosa* Boiss. *, T. orientalis* , and *C. palestina* (collected from labeled location 1, Table 1 ), were significantly different in the number of live adults encountered after treatment compared to the two controls. The former extract had the lowest count (3.00 live adult whiteflis/ plant) and seemed to have the highest repellent effect on the whitefly adults compared to all other treatments in this group. For nymphs, leaf and stem extracts of two plant species, *Verbascum blancheanum* Boiss. And *C. palestina* (collected from labeled location 2, Table 1 ), were significantly different in the number of live nymphs encountered after treatment compared to the two controls. These two, with lowest counts of 16.77 and 17.55 live nymphs per plant (respectively), seemed to have the highest toxic effect on the whitefly nymphs compared to all other treatments in this group. All other extracts were not significantly different in the number of live adults or nymphs from the two controls.

**Table 4.**
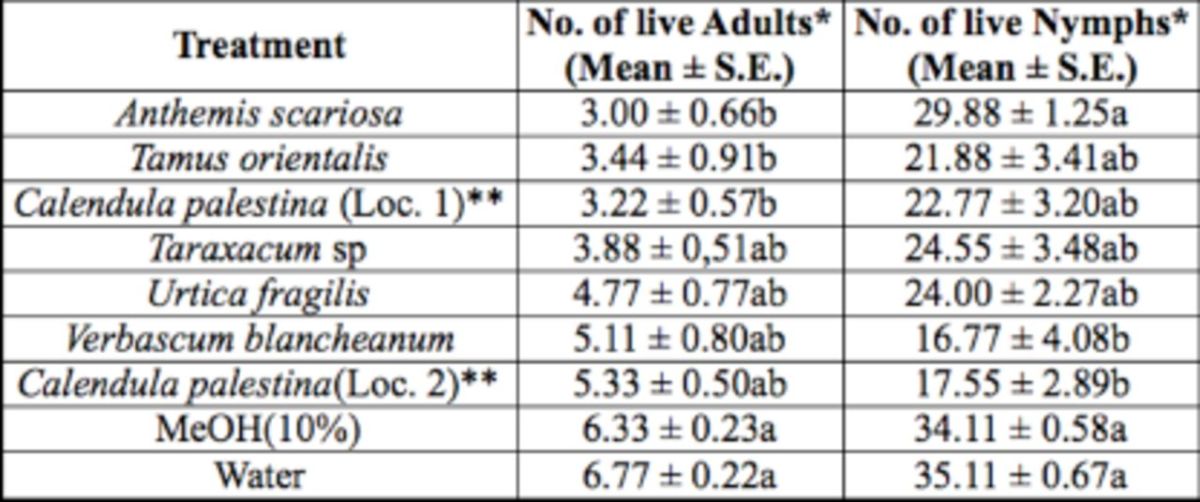
Number of *B. tabaci* adults and nymphs encountered live on treated cucumber plants with methanol leaf and stem extracts of different plant species under controlled glasshouse conditions.

Measurement values presented are mean ± SEM.

*Means in a column followed by different letters (a/b) are significantly different (Student Newman Keuls test, *P* <.05).

**Loc. 1 or 2 refers to location number from where plant species was collected.

In the fourth treatment group, there was a significant difference in the number of live whitefly adults (F = 5.85; df = 5, 53; *P* = 0.0003) and nymphs (F = 6.51; df = 5, 53; *P* = 0.0001) among treatments with four leaf extracts of different plant species and the two controls ( Table 5 ). The leaf extracts of *Verbascum leptostychum* DC., *Heliotropium rotundifolium* Boiss., *Nepeta curviflora* Boiss., and *Salvia rubifolia* Boiss. were significantly different in the number of live adults encountered after treatment compared to the two controls. For the nymphs, only the former two extracts were significantly different in the number of live nymphs encountered after treatment compared to the two controls. *V. leptostychum* extract, which had the lowest count of 3.44 adults and 24.55 nymphs/plant, seemed to have the highest repellent and toxic effects, respectively, to the insect compared to other treatments in this group.

**Table 5.**
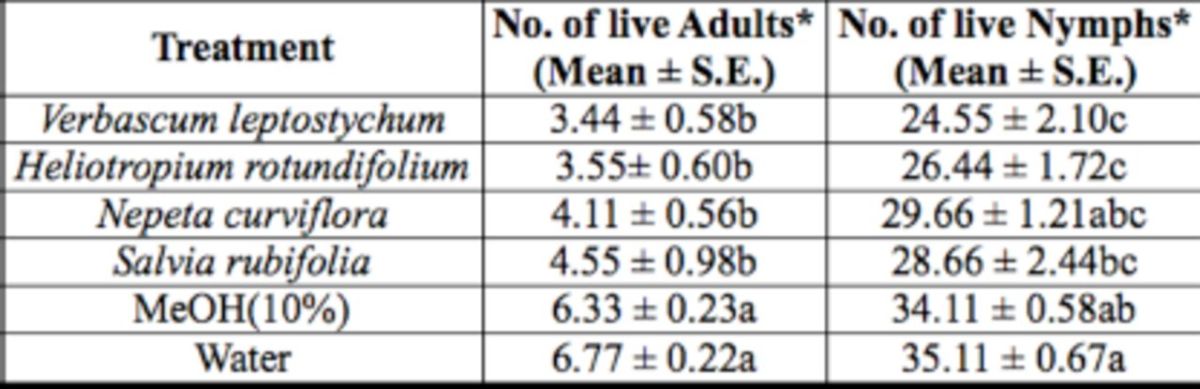
Number of *B. tabaci* adults and nymphs encountered live on treated cucumber plants with methanol leaf extracts of different plant species under controlled glasshouse conditions.

Measurement values presented are mean ± SEM.

*Means in a column followed by different letters (a/b/c) are significantly different (Student Newman Keuls test, *P* <.05).

In the fifth treatment group, comparing different plant parts of one plant species collected from the same location, there was a significant difference in the number of live whitefly adults (F = 2.68; df = 11, 107; *P* = 0.0048) and nymphs (F = 4.90; df = 11, 107; *P* <.0001) among treatments with 10 extracts of different parts of four plant species and the two controls ( Table 6 ). Each plant species was collected from a different location. Leaf and stem extracts of *A. scariosa* resulted in a significantly lower number of adults after treat-treatment compared to the two controls. This extract, which had the lowest count of 3.00 live adult whiteflies/plant, seemed to have the highest repellent effect to the insect compared to all other treatments in this group. However, the lowest number of 17.55 live nymphs/plant was a result of treatment with leaf and stem extracts of *C. palestina* , which was significantly different in the number of live nymphs from all other extracts and the two controls. Similarly, the flower extract of *A. scariosa* was significantly different in the number of live nymphs from the two controls, but it was not significantly different from all other extracts, including the leaf and stem extracts of this *Anthemis* species.

**Table 6.**
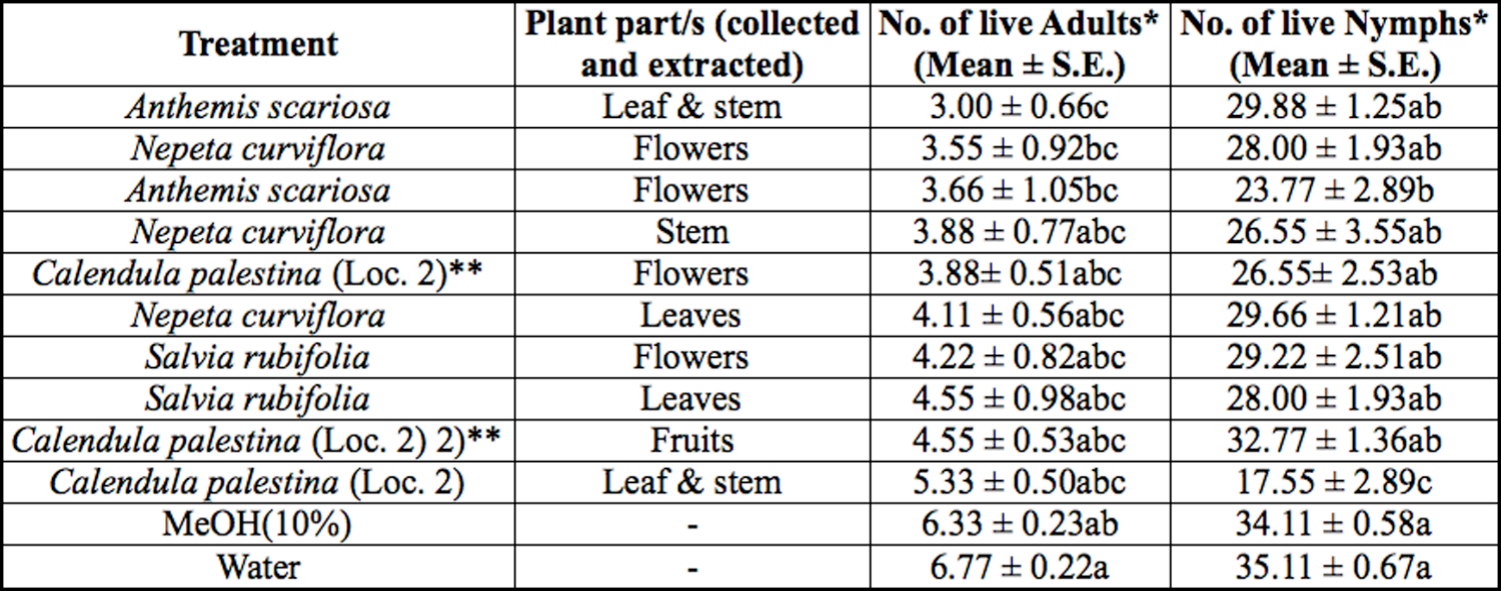
Number of *B. tabaci* adults encountered live on treated cucumber plants, with methanol extracts of different plant parts of one plant species collected from same location, under controlled glasshouse conditions.

Measurement values presented are mean ± SEM.

*Means in a column followed by different letters (a/b/c) are significantly different (Student Newman Keuls test, *P* <.05).

**Loc. 2 refers to location number from where plant species was collected.

For the sixth treatment group, there was a significant difference in the number of live whitefly adults (F = 6.41; df = 7, 71; *P* <.0001) and nymphs (F = 6.65; df = 7, 71; *P* <.0001) among treatments with extracts of similar plant parts of three plant species (collected from different locations) and the two controls ( Table 7 ). All extracts (flower, whole plant, and leaf and stem) of the three plant species collected from their related location (labeled as location 1) resulted in a significantly lower number of adults after treatment compared to the two controls. However, these extracts were comparable in their effect to their counterpart extracts from the other location (labeled as location 2). The flowerextract of *S. pusilla* (collected from labeled location 1, Table 1 ), which had thelowest count of 2.22live adults/plant, seemed to have the highest repellent effect to the whitefly adults compared to all other treatments. For nymphs, the leaf and stem extracts of *C. palestina* , collected from two locations, the whole plant extract of *Achillea damascena* L. (collected from labeled location 1, Table 1 ), and the flower extract of *S. pusilla* (collected from labeled location 2, Table 1 ) were significantly different in the number of live nymphs encountered after treatment compared to the two controls. However, the leaf and stem extracts of *C. palestina* (collected from labeled location 2, Table 1 ), with the lowest count of 17.55 live nymphs/plant, seemed to have the highest toxic effect to the whitefly nymphs compared to all other treatments in this group.

**Table 7.**
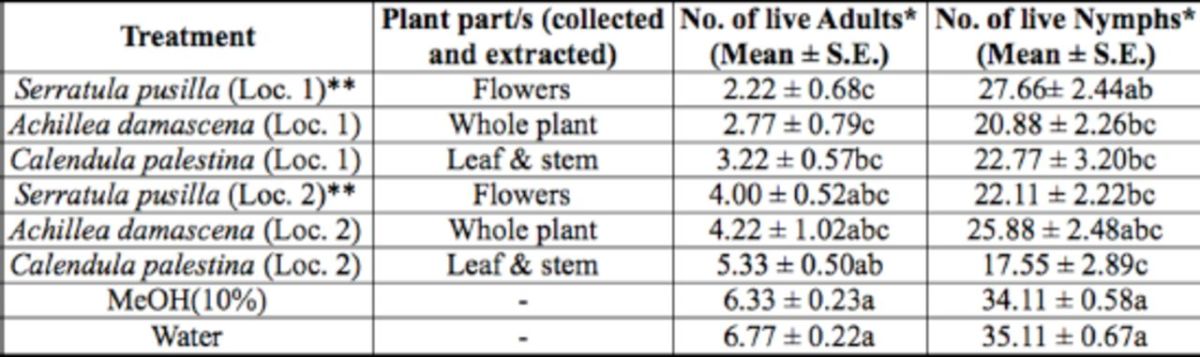
Number of *B. tabaci* adults encountered live on treated cucumber plants, with methanol extracts of same plant part of same plant species collected from different locations, under controlled glasshouse conditions.

Measurement values presented are mean ± SEM.

*Means in a column followed by different letters (a/b/c) are significantly different (Student Newman Keuls test, *P* <.05).

**Loc. 1 or 2 refers to location number from where plant species was collected.

For the seventh treatment group, there was a significant difference in the number of live whitefly adults (F = 12.59; df = 5, 53; *P* <.0001) and nymphs (F = 4.27; df = 5, 53; *P* = 0.0027) among treatments with extracts of the same plant part of four species within two plant genera and the two controls ( Table 8 ). Both whole plant extracts of *Phlomis* sp. and flower extract of *Centaurea ainetensis* Boiss. were significantly different in the number of live adults compared to the two controls. The whole plant extract of *Phlomis damascena* Born., with the lowest count of 2.22 live adults/plant, seemed to have the highest repellent effect to the whitefly adults compared to all other treatments in this group. For the nymphs, the whole plant extrac t of *P. damascena* and the flower extracts of both *Centaurea* spp. were significantly different in the number of live nymphs encountered after treatment compared to the two controls. However, the whole plant extract of *P. damascena* and the flower extract of *Centaurea erengoides* Lam., with the lowest count of 26.55 live nymphs/plant, seemed to have the highest toxic effect to the whitefly nymphs compared to all other treatments in this group.

**Table 8:**
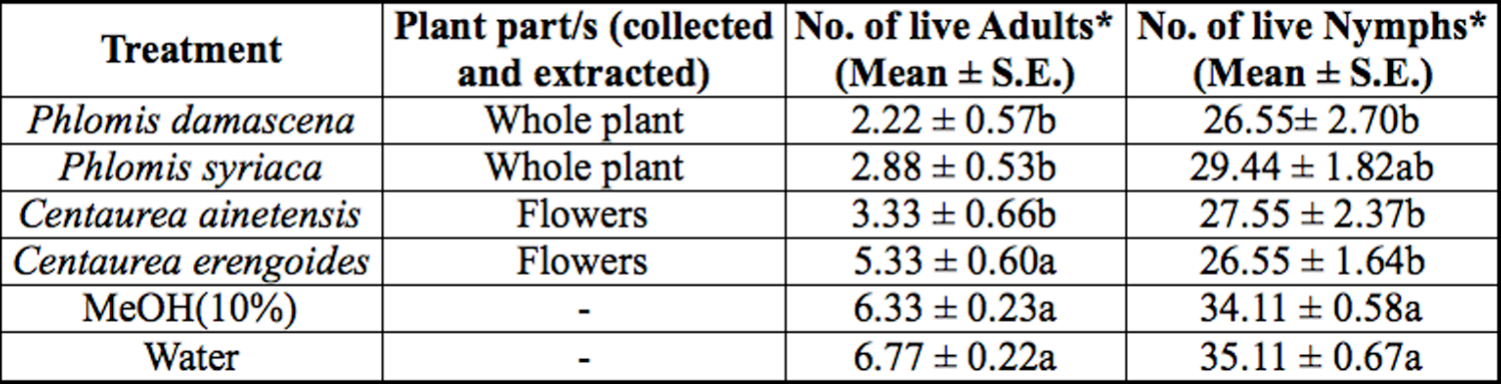
Number of *B. tabaci* adults encountered live on treated cucumber plants, with methanol extracts of the same plant part of different species within same plant genus, under controlled glasshouse conditions

Measurement values presented are mean ± SEM.

*Means in a column followed by different letters (a/b) are significantly different

(Student Newman Keuls test, *P* <.05).

In this study, a commonly observed effect of bioactive extracts was seen in dead nymphs; the body would flatten and turn brown as it dried out. A few bioactive extracts caused oozing, a particular symptom of toxicity on immatures; the nymph’s body was full of dark liquid and the cuticle was soft. The latter symptoms were seen clearly in treatments with whole plant extracts of *Hieracium* sp. L., *Anthemis hebronica* Boiss. & Kotschy, and *A. damascena* (collected from labeled location 1, Table1). Similar symptoms were observed by Jazzar and Abou-Fakhr Hammad (2003) with *Melia azedarach* L. (Sapindales: Meliaceae) fruit extracts on late fourth instar nymphs of *B. tabaci* , in the form of dead nymphs that turned brown due to drying, or from the exudation of body fluids through tissue disruption (“oozing”) after treatment.

## Discussion

A few studies have reported that a number of other species belonging to the same genera of the medicinal plant species tested in this study showed certain effects on insects. This is the first report for the potential effect(s) on survival of insects for the following indigenous medicinal plant species: *A. damascena* , *Al-chemilla diademata* Rothm., *A. hebronica* , *A. scariosa* , *C. palestina* , *C. ainetensis* , *C. erengoides, Cirsium* sp. Boiss., *E.* g *aillardoti* , *H. rotundifolium* , *Hieracium* sp., *Lotus carmeli* Boiss., *N. curviflora* , *O. cynarocephalum* , *P. damascena* , *Phlomis syriaca* Boiss., *R. myosuroudes* , *S. rubifolia* , *S. pusilla* , *T. orientalis* , *V. blancheanum* , and *V. leptostychum* .

In general, the bioactivity of the plant extracts against whitefly might be related to the extracted plant part, the insect instar, the plant species, or the source (i.e., collection location) of the plant material.

In this study, treatment of cucumber plants with methanol extracts of several medicinal plants (either before exposure to adult whiteflies or applied topically to nymph-infested plants) caused a repellent and/or toxic effect against *B. tabaci* , depending on the specific extracted plant part(s). In comparing the bioactivity of extracts of the four major plant parts, 20% and 6.6% of the flower extracts ( Table 2 ), 61% and 30.76% of the whole plant extracts ( Table 3 ), 42.85% and 28.57% of the leaf and stem extracts ( Table 4 ), and 100% and 50% of the leaf extracts ( Table 5 ) were significantly different in number of live adults and nymphs, respectively, from the two controls throughout the experiment. These extracts (except for the leaf and stem extracts of *C. palestina* collected from labeled location 2, Table 6 ) were not significantly different in their effect from each other and from one or more of the other extracts, the latter extracts being comparable in their effects to one or both controls.

Among the 15 flower extracts, the three extracts of *T. orientalis, S. pusilla* (collected from labeled location 1), and *O. cynarocephalum,* which caused significant repellent effect to the adult whitefly, were comparable in their effect to six or more of the remaining 12 extracts, the latter extracts being comparable to the two controls ( Table 2 ). However, the flower extract of *E. gaillardoti,* which significantly lowered the number of live nymphs after topical treatment, was comparable in its effect to all other 14 flower extracts, the latter extracts being comparable to the MeOH(10%) and/or water control ( Table 2 ).

One study involving *Serratula* species other than *S. pusilla* found that ecdysteroids in organic solvent extracts of *Serratula coronata* L. affected biological activities of *Drosophila melanogaster* Meigen ( Odinokov et al. 2002 ). Another study using *Echinops* species other than *E. gaillardoti* found that polythienyl compounds isolated from the roots of *E. grijisii* Hance caused 100% mortality of the first, second, third, and fourth instars of *Aedes aegypti* L. at 0.01, 0.1, and 1 ppm (respectively), while up to 74% mortality of pupae was achieved with 10 ppm of these compounds ( Chang et al. 1990 ).

In comparing the flower extracts of two *Centaur ea* sp., it was found that the flower extract of *C. ainetensis* seemed to be significantly different in its effect from the flower extract of the other species, *C. erengoides,* against the adult whitefly, while the two species were comparable in their significant effect against the whitefly nymphs ( Table 8 ). However, these significant effects were not confirmed when comparing all flower extracts in this study ( Table 2 ). Thus, the difference in the bioactivity of different species within a plant genus is not reflected in this study for the tested plant extracts of *Centaurea* species. However, Barbour et al. (2004) found that the percentage of test microorganisms that were susceptible (20 µL/disc) to the methanol flower extract of *C. ainetensis* was 88.8%; anti-inflammatory bioactivity was also detected in water extracts of this species ( Talhouk et al. 2008 ).

Among the 13 whole plant extracts, the eight extracts of *R. myosuroudes* , *L. carmeli* , *P. damascena* , *A. damascena* (collected from labeled location 1, Table 1 ), *P. syriaca* , *A. diademata* , *Cirsium* sp., and *A. hebronica* caused significant repellent effect to the adult whitefly, but they were comparable in their effect to one or more of the remaining five extracts, except for *R. myosuroudes* , which was significantly different in its effect from four of the remaining extracts ( Table 3 ). However, the whole plant extracts of *R. myosuroudes* , *A. damascena* (collected from labeled location 1, Table 1 ), *A. hebronica* , and *Hieracium* sp., which significantly lowered the number of live nymphs after treatment, were comparable in their effect to one or more of the remaining nine whole plant extracts, except for *R. myosuroudes* , which was further significantly different in its effect from three of these remaining extracts ( Table 3 ). Thus, the methanol whole plant extract of *R. myosuroudes* seemed to exhibit the highest repellent and toxic effects against both adults and second instars. Furthermore, Barbour et al. (2004) found that 88.8% of test microorganisms were susceptible (20 µL/disc) to methanol whole plant extracts of *A. damascena* , *Cirsium* sp., and *R. myosuroudes* .

In one study, ether extracts of *Ranunculus sceleratus* L. caused significant reduction in larval activity, pupal weight, and pupal emergence of fruit flies at all concentrations tested; larval mortality measured at 24 hr after treatment was 100%, 56%, and 16% at 5%, 1%, and 0.05% concentrations, respectively ( Bhattacharyya et al. 1993 ). In another study, *Lotus pedunculatus* Cav. and *L. corniculatus* L. were found to have nematicidal bioactivity ( Molan et al. 2000 ). Furthermore, in species of *Achillea* , *A. fragrantissima* (Forssk) Sch. was one of seven tested plant species that showed high toxicity against *A. aegypti* larvae ( Sathiyamoorthy et al. 1997 ).

In comparing the whole plant extracts of the two *Phlomis* species, *P. damascena* and *P. syriaca* , it was found that these extracts were significantly repellent to adults ( Tables 3 and 8 ), but only the former species caused significant reduction in the number of whitefly nymphs ( Table 8 ). However, these extracts were comparable in their effect to each other against the adult and nymphal instars ( Tables 3 and 8 ). The difference in bioactivity of species within this plant genus is not reflected in this study for the tested plant species of the *Phlomis* genus, similar to the *Centaurea* genus mentioned above; but, the two *Phlomis* species were repellent to adults ( Table 3 ), while the two *Centaurea* species were ineffective against both instars of the whitefly ( Table 2 ). Thus, another type of insect-repelling bioactivity has been detected in our study for the *Phlomis* genus. This genus is known to contain phenylpropanoids that have shown significant cytotoxic, cytostatic, anti-inflammatory, immuno-suppressant, and antimicrobial effects ( Saracoglu et al. 1995 ).

All the seven leaf and stem extracts were comparable in their repellent effect to each other, but only the three extracts of *A. scariosa, T. orientalis,* and *C. palestina* (collected from labeled location 1, Table 1 ) caused significant repellent effects to the adult whitefly ( Table 4 ). For nymphs, the leaf and stem extracts of *V. blancheanum* and *C. palestina* (collected from labeled location 2, Table 1 ), which significantly lowered the number of live nymphs after treatment, were comparable in their effect to the remaining five leaf and stem extracts, except for *A. scariosa,* which was not significantly effective against this instar ( Table 4 ). However, the leaf and stem extracts of the two *Calendula* species from the two locations were comparable in their effects to each other against both the adult and nymph instars ( Tables 4 and 7 ). Bioactivity has been detected in *Verbascum* species other than the *V. blancheanum* and *V. leptostychum* used in this study; purified compounds from an ethanol extract of the dried aerial parts of *V. virgatum* Stokes exhibited anti-germination activity on the seeds of barley, *Hordeum vulgar e* L *.* ( Pardo et al. 2004 ).

In comparing extracts from different parts of a particular plant species, significantly different bioactivity was found only in the leaf and stem extracts of *A. scariosa* and *C. palestina* (collected from labeled location 2, Table 1 ), which were found to be more bioactive against adults and nymphs (respectively) than extracts of other plant parts, such as flowers and fruits (Tables 4, 6, and 7). In previous studies, leaf and fruit extracts of the indigenous tree *M. azedarach* were found to be repellent to whitefly adults, while fruit extracts have shown a significant detrimental effect against nymphal instars of *B. tabaci* ( Abou-Fakhr Hammad et al. 2000 ; Abou-Fakhr Hammad et al. 2001 ; Jazzar and Abou-Fakhr Hammad 2003 ) and *Bemisia argentifolii* (Bellows and Perring) ( Abou-Fakhr Hammad and McAuslane 2006 ). Besides the part of the plant sample studied, other factors may affect the secondary plant metabolites: the maturity of the plant at the collection period of the sample and ecological conditions such as geography, development, hygrometry, and photoperiod during plant growth in different locations. These are important, as any change in these conditions might alter the effectiveness of the plant extract, particularly if the concentrations of the effective metabolites are not constant ( Banthrope and Charlwood 1980 ).

In examining the effect of plant sample location on the bioactivity of the tested extracts, extracts of the same plant parts of *C. palestina, S. pusilla,* and *A. damascena* collected from two different locations were comparable to each other in their bioactivity against the adults and nymphal instars ( Table 7 ). Thus, in our study, no effect of location was observed on the bioactivity of plant extracts from one species collected from different sites. This could be attributed to the fact that the two collection locations for a particular species were not geographically distant from each other. For example, plant samples for leaf and stem extracts of *C. palestina* were collected from two sites in one location, Ibel El Saki ( Table 1 ), located in the Marjaayoun-Nabatieh District. Plant samples for *S. pusilla* flower extracts were collected from Mahmiet Baal-beck and Aineta ( Table 1 ); both locations are in Baalbek District. Samples of *A. damascena* for whole plant extracts were collected from two locations (Table1): Hasroun, located in the Bsharri District in the North Governorate of Lebanon, and Rassem el Hadath, located in Baalbek District in the Beqaa Governorate of Lebanon (the Baalbek District is located east of the Bsharri District.) In certain studies, the location or site factor could be related to the phenomenon reported by Arias and Hirschmann (1988), who confirmed that production of azadirachtins might suffer from strong bio-geographic dependence. It has been also reported that crude extracts of *M. azedarach* from Paraguay were devoid of any anti-molting activity, whereas activity was found in Kenyan *M. azedarach* ( Morgan and Thornton 1973 ).

The four leaf and stem extracts of *V. leptostychum, H. rotundifolium, N. curviflora,* and *S. rubifolia* seemed to be comparable in their repellent effect on adults ( Table 5 ). For nymphs, the leaf extracts of *V. leptostychum* and *H. rotundifolium* significantly lowered the number of live nymphs after treatment and were comparable in their effect to the remaining two leaf extracts ( Table 5 ). Furthermore, the leaf extracts of *N. curviflora* and *S. rubifolia* were comparable in their effect to the extracts of other plant parts, such as flowers and stems, against the adult and nymphal instars ( Table 6 ). However, all these extracts were not effective against the two whitefly instars ( Tables 2 and 6 ). This verifies that the tested extracts of different plant parts of *N. curviflora* and *S. rubifolia* were comparable in their bioactivity and were not effective against adult and nymphal *B. tabaci.* However, Barbour et al. (2004) found that 88.8% and 99.9% of test microorganisms were susceptible (20 uL/disc) to methanol extracts of *N. curviflora* leaf and stem parts, respectively, and 99.9% were susceptible to *V. leptostychum* flower extract.

*Heliotropium* species other than the *H. rotundifolium* leaf and flower extracts tested in our study have shown different insecticidal effects. *H. bacciferum* Forskal deterred oviposition in the cowpea bruchid, *Callosobruchus maculates* Fabricius ( Elhag 2000 ). Isolated pyrrolizidine alkaloids from *H.*

*floridum* Gay showed that 3′- acetyltrachelanthamine is a strong antifeedant with low toxicity against the Colorado potato beetle, *Leptinotarsa decemlineata* ( Reina et al. 1998 ). Furthermore, resin exudates of *H. huascoens* e I.M. Johnston were found to show antifeedant and nutritional effects on *L. decemlineata* adults ( Villarroel et al. 2001 ). Bioactivity was detected in *Nepeta* species other than the extracts of *N. curviflora* tested in this study; essential oils of catnip, *N. cataria* L., caused ceasing of tunneling by termites, *Reticulitermes flavipes* Kollar and *R. virginicus* Banks (Isoptera: Rhinotermitidae), causing limited mortality, which indicated that the termites avoided the treated sand ( Peterson and Ems-Wilson 2003 ).

*Salvia* species other than the flower and leaf extracts of *S. rubifolia* tested in this study also caused some effects against a series of insects. Plant material of *S. dominica* L. powdered and extracted with 3:1 acetone at 30°C produced larvicidal effects on the fourth instars of *A. aegypti* at a dose of 500 ppm and on fifth instars of *Spodoptera littoralis* at a dose of 200 µg/cm ^2^ . Counts were taken at 24 hr and the mortality observed was above 80%. Essential oils of *S. dominica* were also found to be active against *Oryzaephilus surinamensis* L. (Cucujidae) at a concentration of 15µL/L, and 55% mortality was recorded ( Shaaya et al. 1991 ).

*Salvia* species other than the flower and leaf extracts of *S. rubifolia* tested in this study also caused some effects against a series of insects. Plant material of *S. dominica* L. powdered and extracted with 3:1 acetone at 30°C produced larvicidal effects on the fourth instars of *A. aegypti* at a dose of 500 ppm and on fifth instars of *Spodoptera littoralis* at a dose of 200 μg/cm2. Counts were taken at 24 hr and the mortality observed was above 80%. Essential oils of *S. dominica* were also found to be active against *Oryzaephilus surinamensis* L. (Cucujidae) at a concentration of 15μL/L, and 55% mortality was recorded ( Shaaya et al. 1991 ).

In comparing other extracts of different plant parts of a single plant species collected from same location, it was found that the leaf and stem extracts and flower extract of *A. scariosa* were comparable in their repellent and toxic effects against adult and nymphal whiteflies ( Table 6 ). However, the leaf and stem extracts caused a significant repellent effect against adults ( Tables 4 and 6 ), while the flower extract caused a significant toxic effect against nymphs ( Table 6 ). However, the latter extract did not cause any effect against adults and nymphs when compared with other flower extracts in this study ( Table 2 ). On the other hand, the extracts of *C. palestina* (collected from labeled location 2), including the flower extract, fruit extract, and leaf and stem extracts, were comparable in their effects against adults and nymphs, except for the leaf and stem extracts, which were significantly effective against the nymphs compared to all other treatments ( Tables 4 and 6 ). Thus, the leaf and stem extracts of *A. scariosa* and *C. palestina* (collected from labeled location 2) were more bioactive than extracts of other plant parts of the two species. Bioactivity was detected in species other than the *A. scariosa* and *A. hebronica* tested in this study. Volatile fractions from leaves of *A. melampodina* Delile have caused marked larvicidal activity against *Culexpipiens* L. with LC50 of 71.86 ppm ( Grace 2002 ). *In vitro* antibacterial activity was also detected in lactones isolated from *A. altissima* ( Konstantinopoulou et al. 2003 ).

The effect of some plant extracts against only one of the whitefly instars might be related to the ability of the insect instar to overcome the bioactivity of the extract either physiologically or behaviorally. It could also be related to the volatility of the extract; usually, the adult whitefly is more severely affected than the sessile second nymphal instar, which is covered by its scale-like cuticle. In our study, the following plant extracts caused significant repellent effect against the adult whitefly, but they were ineffective against the nymph: flower extracts of *T. orientalis* , *S. pusilla* (collected from labeled location 1, Table1), and *O. cynarocephalum;* whole plant extracts of *L. carmeli* , *P. syriaca* , *A. diademata* , and *Cirsium* sp.; leaf and stem extracts of *A. scariosa* , *T. orientalis* , and *C. palestina* (collected from labeled location 1, Table1), and leaf extract of *N. curviflora.* However, the following extracts caused significant toxic effects against the nymphs, but were ineffective against the adults: flower extract of *E. gaillardoti,* whole plant extract of *Hieracium* sp., and leaf and stem extracts of *V. blancheanum* and *C. palestina* (collected from labeled location 2, Table 1 ). Furthermore, a few extracts were highly effective against both adults and nymphs: whole plant extracts of *R myosuroudes, A. damascena* (collected from labeled location 1, Table 1 ), and *A hebronica;* and leaf extracts of *V. leptostychum* and *H. rotundifolium.*

Other factors, such as seed drying, exposure to sunlight, storage, and the method of extraction, also contribute to the effect of active ingredients in plant extracts ( Ermel et al. 1984 ). A search for suitable organic solvents to extract insecticidal secondary compounds from the plant is always needed. Abou-Fakhr Hammad et al. (2000) found that *M. azedarach* methanol extracts were more active against *B. tabaci* than extracts with other solvents, such as acetone, ether, and water. Similarly, neem methanol extracts were reported to be 50 times more concentrated than water extracts that were effective as pesticides (Anon. 1992). Furthermore, pentane extracts of neem seed kernels proved to cause a higher mortality rate in female adult mites than the methanolic extract, which was not as efficient. On the basis of efficiency, the extracts can be correlated with the dielectric constants of the extracting solvents: pentane, 1.8; chloroform, 4.8; butanol, 17.1; acetone, 20.7; methanol, 32.6; and water, 78.5 ( Kennedy and Van Impe 1995 ). Plant extracts might also have low residual stability on plants; insecticides of biological or botanical origin are especially susceptible to UV degradation, resulting in reduced efficacy ( Sundaram et al. 1995 ). Thus, prolonged application of the botanical insecticidal extract on foliage was found to increase the pesticide effectiveness and to enhance its biological activity ( Sundaram 1996 ).

We conclude that 22 out of 41 medicinal plant extracts were found to have potential bioactivity against adult and/or nymphs of *B. tabaci.* The effect of these bioactive extracts might be enhanced by selecting other organic solvents for extraction or by increasing the concentration of the active ingredient of these extracts, as long as this does not prove phytotoxic to the tested plants. Furthermore, the most bioactive extracts of *R. myosuroudes* and *P. damascena* against whitefly adults and/or nymphs were further fractionated for identification of their chemical groups with potential pesticidal bioactivity against *B. tabaci.*
